# Tropical Legume Crop Rotation and Nitrogen Fertilizer Effects on Agronomic and Nitrogen Efficiency of Rice

**DOI:** 10.1155/2014/490841

**Published:** 2014-05-22

**Authors:** Motior M. Rahman, Aminul M. Islam, Sofian M. Azirun, Amru N. Boyce

**Affiliations:** Institute of Biological Sciences, Faculty of Science, University of Malaya, 50603 Kuala Lumpur, Malaysia

## Abstract

Bush bean, long bean, mung bean, and winged bean plants were grown with N fertilizer at rates of 0, 2, 4, and 6 g N m^−2^ preceding rice planting. Concurrently, rice was grown with N fertilizer at rates of 0, 4, 8, and 12 g N m^−2^. No chemical fertilizer was used in the 2nd year of crop to estimate the nitrogen agronomic efficiency (NAE), nitrogen recovery efficiency (NRE), N uptake, and rice yield when legume crops were grown in rotation with rice. Rice after winged bean grown with N at the rate of 4 g N m^−2^ achieved significantly higher NRE, NAE, and N uptake in both years. Rice after winged bean grown without N fertilizer produced 13–23% higher grain yield than rice after fallow rotation with 8 g N m^−2^. The results revealed that rice after winged bean without fertilizer and rice after long bean with N fertilizer at the rate of 4 g N m^−2^ can produce rice yield equivalent to that of rice after fallow with N fertilizer at rates of 8 g N m^−2^. The NAE, NRE, and harvest index values for rice after winged bean or other legume crop rotation indicated a positive response for rice production without deteriorating soil fertility.

## 1. Introduction

Rice is the most widely consumed staple food and most commercially important crop for more than 3 billion people in the world's human population [1]. Nitrogen is quantitatively the most essential nutrient for plants [2] and a major constraint and contributing factor for low productivity and widespread food insecurity in most rice-based cropping systems in Asia [3, 4]. The intensive cultivation of cropping practices with high yielding rice varieties requires better soil and nutrient management [5]. Alternatively, long-term cropping can degrade soil fertility [6]. Soil texture and crop rotation practices can influence rates of N fertilizer application in rice crop [7, 8]. However, imbalanced rates and injudicious methods of fertilizer application can lead to poor N efficiency, N losses due to leaching, and other chemical and biological processes in soil [9–12], resulting in a series of environmental hazards and economic loses. Thus, it is obvious that poor nitrogen use efficiency (NUE) causes higher production costs and induces lower net returns for rice growers [13]. So, an efficient N utilization must be ensured for sustainable crop production for the benefit of environment and economic reasons [14, 15]. Optimum use of N fertilizer is a crucial step to improve NUE, while a positive relation between soil N supply and crop N demand is one of the key factors to appropriate N utilization by plants [16].

Soil N supply through biological nitrogen fixation (BNF) by associated microbial populations is one of the principal sources of N for rice production. However, the loss of soil N occurs continuously through removal of plant or harvesting of grain and chemical processes of the soil [12]. In addition, the indigenous soil N supply in wetland rice may decline with intensive rice cultivation unless it is restored by BNF [17]. Considering both environmental and economic perspectives, maintenance of native soil N resource and improvement of N output from plant sources are one of the desirable options to reduce the use of chemical fertilizer in rice cropping system [18]. Soil N loss may be minimized by using effective legume crops which can supply sufficient BNF input to enhance soil N by improved recycling of N through plant residues [19]. Thus, the combined indigenous soil N and N achieved through legume in BNF have the potential for N enrichment in soil which will increase NUE of crops and total N output in a lowland rice-based cropping system [20]. In addition to fixing atmospheric nitrogen, leguminous green manures play a significant role in conserving NO_3_ [21, 22]. It is well recognized that the legume plant can add organic N and their residues contribute to the improvement of soil texture and microbial activity. However, rice growers believe that the accrued N benefits will vary among different legume systems [23].

In Malaysia, most of the rice growing areas are well established with irrigation systems and farmer's practicing double cropping systems with high yielding varieties of rice crop [5], which mainly depends on inorganic N fertilizer and other chemical fertilizers. The government has spent more than 3.0 billion US$ (RM 9.2 billion) to import 4.2 million tons of mineral fertilizers to sustain crop production in Malaysia [24]. Subsequently, its policy has focused more towards agroecological, healthier, and sustainable food production practices through an integrated approach of rice cultivation with crop rotation using vegetable, legume, and intercropping practices for sweet corn, maize, and organic farming to minimize the dependence on mineral fertilizers [25–27]. Inclusion of grain legumes or green manure legume crops in rotation with rice or corn can protect degradation of soil fertility [28–30], improve soil structure, water holding capacity [31], and result in greater productivity and higher income, while minimizing production risk and ensuring long-term sustainability, as well as ensure a greener environment [30, 31]. Incorporation of crop residues alters the soil environment that in turn influences the microbial population's activity in the soil and subsequent nutrient cycle [23] and will sustain rice productivity through replenishing soil organic matter [32]. The presence of soil organic matter is a key indicator of soil quality, which provides plant nutrients upon mineralization and eventually improves soil properties [33].

Legume crop residues contribute to organic N and after decomposition by soil microbes, through mineralization, add available N for the next crop [34], and ameliorate the nutrient status of the soil. In Malaysia, rice growers cultivate two rice crops per year and sometimes five crops in a two-year period, but crop rotation practices of rice with tropical grain legumes or green manure legume crops are not often used [27]. This has brought about soil fertility deterioration, which threatens the ecosystem through intensive application of inorganic chemical fertilizers. Thus, the appropriate management and efficient utilization of crop residues are important for the proper amendment of soil quality and crop productivity under a rice-based cropping system in the tropics [22]. Indeed, the use of grain legumes or cover crops has been proven to be commendable in terms of its positive effects. Bush bean, long bean, sprouted mung bean seed, and winged bean are traditional vegetables cultivated in marginal land and there are ample opportunities to adapt these crops in an upland rice-based crop rotation system. Presently, growers are concerned about the quality and as well sustainable use rather than the quantity of food production. Attention has been focused towards the use of legume crops to improve soil health for productivity of rice crop. The practice of using tropical legumes such as, bush bean, long bean, winged bean, or mung bean, alone or in combination with inorganic N fertilizers, offers promising scope as an N supplement to rice crop rotation systems. No systematic research has been carried out on the consequence of N in green manure legume and the productivity of legume crops and their effects on soil N dynamics and contributions to the yield and N uptake of the following rice crop in Malaysia. The combined effect of legume residues and indigenous nutrient supplies or fertilizer uptake and losses in rice-based systems is little understood. The present study was undertaken to assess the addition of legume residues to plant N uptake, NAE, and NRE and also the amount of fertilizer N essential for optimizing rice yield when legumes are enclosed in the system.

## 2. Materials and Methods

### 2.1. Experimental Plan and Management

The experiments were carried out at the greenhouse, University of Malaya, Kuala Lumpur, Malaysia, during 2010 and 2011. The clay loam soil was collected at depth of 30 cm from rice field in Selangor (1° 28′ 0′′ N, 103° 45′ 0′′ E), Malaysia. No specific permits were required for the described studies and no specific permissions were required for these activities. In addition, the locations were not protected and the studies did not involve endangered or protected species. The soil used had the following chemical properties: pH 6.55 ± 0.20 (1 : 5 w/v water), CEC 15 (cmol_c_ kg^−1^ soil), organic C 1.75 ± 0.48% (CHNS analyzer, model NA 1500), total N 0.18 ± 0.04%, NH_4_-N 6.37 ± 1.25 (mg 100^−1 ^g soil), exchangeable CaO 171.0 ± 21.15 (mg 100^−1 ^g soil), exchangeable MgO 10.8 ± 2.75 (mg 100^−1 ^g soil), and exchangeable K_2_O 14.9 ± 9.06 (mg 100^−1 ^g soil). The soils were thoroughly mixed and unwanted inert materials were discarded through sieve (2 mm mesh) to produce homogenous soil composites. The experimental pots (height 46 cm × diameter 54 cm = surface area 1 m^2^) were filled with soil up to about 36 cm height (height 36 cm × diameter 54 cm = surface area 0.84 m^2^) of each pot. All data was converted into 1 m^2^. The seeds of bush bean (*Phaseolus vulgaris* L.), long bean (*Vigna unguiculata* (L.) Verdc.), mung bean (*Vigna radiata* (L.) R. Wilczek), winged bean (*Psophocarpus tetragonolobus* (L.) D.C.), and corn (*Zea mays* L.) were sown in moistened soil to ensure germination. All legume crops were fertilized with N fertilizer (urea 46% N) at rates of 0, 2, 4, and 6 g N m^−2^ while HYV corn and HYV rice fertilized at rates 0, 4, 8, and 12 g N m^−2^ in the first cycle of the experiment in 2010. Fertilizer was applied in soil prior to the sowing of seeds of bush bean (cv-MKB 1), long bean (cv-MKP 5), mung bean (cv. local), winged bean (cv. local), and corn. Corn was used as non-N_2_-fixing reference plant for estimation of N_2_ fixation by N difference method (NDF). In addition, 16 fallow pots were assigned to fulfill the requirement of rice after fallow crop rotation. Each crop was tested in an individual experiment and each experiment was conducted under completely randomized design with four replications, which covered 16 pots for each crop and a total of 96 pots for all the crop cycles. Rice was transplanted as the 2nd crop after harvesting of first crop cycles of corn and or incorporation of legume residues. After completion of the 1st cycle of rice crop, all legumes and corn were grown in the same pot as third crop in the 2nd cycle. No N fertilizer or other chemical fertilizers were applied in the 2nd year to estimate the enduring effect of legume residues for the next crop. Concurrently, fallow pots were used as rice after fallow crop rotation cycle. Bush bean, long bean, mung bean, winged bean, and corn were planted in early March of 2010 and 2011. All legume crops and corn were harvested at 70 days after emergence. Rice seedlings (14 d old) were transplanted during the 2nd week of July for both years. In the 1st cycle, the rice crop was fertilized in three stages: one third before transplanting, one third at tillering, and one third at panicle primordial initiation stages, respectively. Rice was harvested at the stage of physiological maturity during the second week of November in both years.

Above ground plant parts of all legumes were harvested and fragmented into small pieces and spread into the pots and incorporated to a depth of about 8–10 cm into soil with mulching. This was followed by watering and the pot was left stagnant for 30 days to prepare for rice transplanting. Water was applied every alternate day to keep soil moist until physiological maturity of rice plant.

### 2.2. Determination of Total Dry Matter and Nitrogen

After harvesting of each crop randomly, 200 g fresh plant samples were taken and dried to constant weight at 70°C. Dry weight was measured by digital sensitive balance and converted into above ground biomass yield, rice grain yield, and N content for each crop per unit area. Biomass and grain yield were determined from each pot. Total N concentration was determined by micro-Kjeldahl digestion method [34, 35].

### 2.3. Estimation of Biological Nitrogen Fixation and Nitrogen Use Efficiency (NUE)

It is well documented that soil and fertilizer are the sources of N for nonfixing crops while sources of N for fixing crops (F) are from the soil, fertilizer, and atmosphere. For nonfixing and fixing crops, the proportions of N from all the available sources can be denoted as follows [36]:

(1) $$\begin{array}{c} \%{\text{Ndff}}_{\text{NF}}+\%{\text{Ndfs}}_{\text{NF}}=100\%,\\ \%{\text{Ndff}}_{\text{F}}+\%{\text{Ndfs}}_{\text{F}}+\%{\text{Ndfa}}_{\text{F}}=100\%,\\ \%\text{Ndfa}=100-\left(\%{\text{Ndff}}_{\text{F}}+\%{\text{Ndfs}}_{\text{F}}\right), \end{array}$$

where Ndff_NF_ denotes N derived from fertilizer for nonfixing crops, Ndfs_F_ denotes N derived from soil for nonfixing crops, Ndff_F_ denotes N derived from fertilizer for fixing crops, Ndfs_F_ denotes N derived from soil for fixing crops, and Ndfa_F_ denotes N derived from atmosphere for fixing crops.

The N difference method (NDF) was used to estimate the contributions of BNF to total N accumulation in the legumes [37]. Consider

(2) $$\begin{array}{c} \%\text{Ndfa}=100\frac{\left[\left(\text{Legume}\;\text{N}-\text{Reference}\;\text{N}\right)\right]}{\left(\text{Legume}\;\text{N}\right)}. \end{array}$$

The following N-efficiency parameters were calculated for each treatment [38–40].

N agronomic efficiency (NAE g g^−1^) = (grain yield at N*x* − grain yield at N0)/applied N at N*x*, and N fertilizer recovery efficiency (NRE %) = (N uptake at N*x* − N uptake at N0)/applied at N*x*.

Data were analyzed following analysis of variance [41] and treatment means were compared based on the least significant difference (LSD) test at the 0.05 probability level.

## 3. Results and Discussion

### 3.1. Biomass Yield and Nitrogen Uptake of Legume Crops

Legume crop biomass yield and N uptake were influenced significantly with N fertilizer application. Winged bean grown with N fertilizer at rates of 4, 8, and 12 g N m^−2^ produced greater biomass (>185 g m^−2^) and N uptake (>6.5 g m^−2^) for both years. In 2010, the biomass yield of bush bean was 148–170 g m^−2^ (Table 1) with a parallel N accumulation of 5.0–5.9 g m^−2^. As shown in Table 2 in 2011, the biomass of bush bean was 140–163 g m^−2^ with the comparable N accumulation of 4.7–5.6 g m^−2^ (Table 2). Biomass yield and N uptake of bush bean, long bean, and mung bean were slightly lower compared to winged bean. Biomass and N uptake was higher in winged bean compared to the other tested legume crops in both years. All the tested legume crops, other than winged bean, recorded consistently identical biomass yield and N uptake. Nitrogen uptake in the different legume species vary according to the influence of biomass production and N content in the plant tissues. Earlier studies have reported that faba bean produced >10 kg biomass m^−2^ which recorded >35 g N m^−2^ [33]. Other studies have also reported that N uptake by cover crops produced 4.5 to 22.5 g of N per m^−2^ [42–44]. Furthermore, total N accumulation produced by* Vicia faba* L. plant residues were lower (11.6 to 19.9 g m^−2^) than those gained by* Vicia villosa* Roth (16.4 to 26.4 g m^−2^) [23, 44]. In this study, N uptake by winged bean plant residues was apparently higher than other crops. This was probably due to the biomass yield of long bean, bush bean, and mung bean being lower than that of winged bean.

### 3.2. Nitrogen Fixation and Nitrogen Recovery Efficiency of Legume Crops

Nitrogen fixation and NRE of legume crops were significantly affected by the use of N fertilizer. Nitrogen fixation in winged bean was 22–42% of the total plant N, 10–29% in bush bean, 6–25% in long bean, and 3–24% in mung bean in 2010. Irrespective of N fertilizer used in 2010, N_2_ fixation in winged bean was 30–41%, 13–29% in bush bean, 10–25% in long bean, and 9–24% in mung bean in 2011 as determined by the total NDF method (Table 3). The highest N_2_ fixation was achieved by winged bean (41-42%) followed by bush bean (29%), long bean (24-25%), and mung bean (22–24%) when all the legume crops were grown without N fertilizer for both years. Earlier studies have shown that N_2_ fixation in* Cajanus cajan* (L.) Millsp. was 44 to 95% [43] while N_2_ fixation by* Vicia faba* and* Vicia villosa* was 41% and 78% of total plant N, respectively [44, 45].

Winged bean grown with 2 or 4 g N m^−2^ obtained appreciably higher NRE in 2010, while NRE was remarkably higher (29%) when winged bean was grown without N fertilizer in 2011 (Table 4). The superior N_2_ fixation and NRE of all tested legume crops were directly linked to the lower rate of N fertilizer application. Nitrogen recovery efficiency in long bean was lower in 2011. The lowest NRE was recorded by legume crops treated with the highest rate of N fertilizer used to the preceding rice crop. Our findings have shown that legume residues mixed into rice crop rotation enrich not only to raise yield but also to nurture and ameliorate soil fertility by virtue of their ability to add ample quantities of atmospheric N. Legumes can make a significant contribution to advance soil fertility and improve soil texture [46–48]. Biomass yield, legume N demand, the capacity to fix N_2_, and adaptability to specific environments are important attributes for BNF [49]. Nitrogen content in legume above ground biomass ranged from 4.5 g N m^−2^ to 6.9 g N m^−2^, which was integrated into the soil. Among the tested legume plants, winged bean supplied substantially higher N over their residues in each season. The higher quantities of nitrogen N_2_ secured in winged bean came from its larger supply of biomass production as well as a higher number of nodules.

### 3.3. Biomass Yield of Rice after Legume Crops

Biomass yield of rice was strongly influenced by the treatment variables. Significantly greater biomass production was produced by rice after winged bean with 4, 8, and 12 g N m^−2^ in both years (Table 5). Biomass yield was lower when rice was rotated with other legume crops than in rice after winged bean rotation, but it was higher than in rice after corn rotation. Rice after fallow with 8 and 12 g N m^−2^ also produced appreciably greater biomass and it was almost at par with rice after bush bean, rice after long bean, or mung bean although the level was comparably lower than rice after winged bean. The lowest biomass yield was produced by rice after corn or rice after fallow grown without N fertilizer (Table 5). The accumulated biomass following rotation with winged bean is possibly an indication of N contribution from the above and below ground residues of the legume. On the contrary, a considerable amount of urea was used and apparently volatile loss of ammonia occurred in rice after fallow rotation with fertilizer. A possible reason this was due to the N fertilizer applied up to panicle initiation stage. Earlier studies have suggested that an appreciable amount of N can be lost via ammonia volatilization when urea was applied at sampling stage just before top dressing [50]. The presence of soil organic matter and decomposed plant residues are the primary determinants of total plant-available N supply for plant growth which is controlled by the balance between N immobilization and mineralization as mediated by soil biota as well as the contributions from applied organic and inorganic N sources and losses from the plant-available N pool [51].

### 3.4. Nitrogen Uptake and Nitrogen Efficiency of Rice after Legume Crops

Total N uptake was influenced by N fertilizer application in each crop rotation (Table 6). Maximum N uptake was observed in rice rotation with winged bean with 4, 8, and 12 g N m^−2^. Rice after long bean or bush bean or mung bean with 8 and 12 g N m^−2^ obtained appreciably greater N uptake compared to rice after fallow or corn. Rice after fallow or in rotation with corn grown without N fertilizer application recorded the least N uptake (Table 6). The increased plant N following rotation with winged bean could probably be attributed to the N contribution from the addition of larger amounts of above ground plant parts and below ground plant residues of legume. The quality of residues in legume plants in rotation with rice crops might be the reason for the greater N uptake compared to rice after corn and rice after fallow rotation. Despite the smaller below ground residues of other pulses other than cereals and other crops, they possess a higher microbial population in the soil which influences the concentrations of nutrients released in the rhizosphere for N uptake by the plants [52].

Among crop rotations, rice after winged bean with N at rates of 4, 8, or 12 g m^−2^ showed the highest N uptake, whilst rice after other legumes with N at rates of 4, 8, or 12 g m^−2^ recorded intermediate N uptake and rice after corn and rice after fallow rotation recorded the lowest. Soil microbial population increased rapidly when young and relatively succulent green manure crop are incorporated into the soil. The soil microorganisms multiply faster to invade the freshly incorporated plant residues. After decomposition of plant residues through microbial breakdown, nutrients remain within the plant tissues and nutrient rich dead microbes, which are released and made available to the following crop [51]. In our study, all legume crops were added into soil at the pod formation stage. Apparently, this could slightly slow the breakdown of legume residues resulting in poor volatile loss of ammonia during rice after winged bean or rice in rotation with other legume growing cycles. While in rice fallow systems with fertilizer, a substantial amount of the applied urea are seemingly lost by ammonia volatilization.

Nitrogen recovery efficiency (NRE) was influenced significantly by N fertilizer application in each crop rotation (Table 7). The highest NRE (32.5%) was obtained by rice after winged bean with 4 g N m^−2^. In this study, the observed NRE values were relatively lower than those reported in previous studies where N fertilizer was used later in the crop growing period. The NRE was significantly higher in 2010 than in 2011, possibly due to the fact that the highest grain yield was recorded in 2010 (Table 7). The higher grain yield of rice in 2010 could be due to higher N uptake, caused by both legume residual effects along with N fertilizer. Regardless of fertilizer, rice after legumes obtained higher NRE than rice after fallow or corn in both years. In India, the large variation in NRE (18%—1st year and 49%—2nd year) was observed in rice-wheat systems. This difference was directly linked with poor yields in the first year caused by unfavorable weather and highlights the importance of higher crop growth and yield to higher NRE [49]. Higher NRE values were reported in rotation than in monoculture [40, 53]. Regardless of N fertilizer rate, the NRE values were 15–33% in 2010 and 13–30% in 2011 for rice after winged bean (Table 7). The NRE values were 20–29% in 2010 and 20–28% in 2011 for rice after bush bean or long bean or mung bean. In our study, the NRE values were lower compared to NRE values (42%) obtained in developed countries [50]. A possible reason could be due to the fact that the present study was carried out under greenhouse conditions which does not show the full potential of legume performance while in developed countries studies were conducted in on-station field conditions.

The higher N recoveries in legume rotations could be due to the enrichment of soil N. The below ground pool of legume N is an important source of N for subsequent crops [51]. When the soil N content rises, the amount of sequestered N contributes to a greater NUE of the cropping system and the amount of sequestered N achieved from applied N results in a higher NRE [51]. The average NUE gained by rice farmers is 31% of applied N based upon on-farm estimation in the major rice production countries of Asia. In contrast, it is documented that the recovery efficiency of nitrogen for rice normally varies between 50 and 80% in well-managed field experiments [51]. Therefore, emphasis has been given to improve recovery efficiency of nitrogen because the N fertilizer is the greatest source of N input and loss from cereal cropping systems.

Nitrogen agronomic efficiency (NAE) was affected significantly by N fertilizer application in each crop rotation. Rice after winged bean grown with 4 g N m^−2^ recorded the highest NAE (24 g g^−1^ to 27 g g^−1^) for both years (Table 8). Rice after long bean with 4 g N m^−2^ also showed a similar trend although NAE was lower than rice after winged bean systems regardless of N fertilizer for both years (Table 8). The NAE trends were similar in both 2010 and 2011. Rice after fallow and rice after long bean with 4 g N m^−2^ also showed similar trends although NAE credentials of rice after winged bean systems indicate a positive response to rice production without deteriorating soil fertility. Therefore, the nitrogen agronomic efficiency and grain yield was significantly increased by the legume plant residues when supported by organic sources of N. The results showed that the increase in the application of N caused the decline of agronomic nitrogen efficiency. The highest and the lowest agronomical nitrogen use efficiencies were obtained in the 60 and 180 kg N ha^−1^ which showed that NAE decreased with the increasing rate of N fertilizer used [54]. From this, it can be said that the fertilizer response to NRE was poor but grain yield and N uptake showed significant differences among fertilizer rates. In this regard, NUE was reduced at higher rates of fertilizer N rate, possibly due to greater losses from soil via volatilization or leaching losses [53]. With respect to fertilizer utilization in rice crop, current N fertilizer management strategies must be improved in upland as well as wetland rice growing areas in Malaysia.

### 3.5. Grain Yield and Harvest Indices of Rice

Rice grain yield increased significantly by the amendment of soil with addition of legume residues and N fertilizer application (Table 9). Maximum grain yield (603–684 gm^−2^) was produced by rice after winged bean with 4, 8, and 12 g N m^−2^ in both years. Rice in rotation with corn (293–349 g m^−2^) and rice after fallow (342–371 g m^−2^) grown without fertilizer recorded the lowest yield of rice crops. Rice after long bean or bush bean or mung bean with 8 and 12 g N m^−2^ gave similar yields for both years. In 2011, a slightly lower yield was obtained but a similar trend was observed in all the crop rotation systems. Rice in rotation with corn grown with 8 g N m^−2^ and 12 g N m^−2^ produced comparatively lower yield than other counterparts. Identical yield was observed in rice after fallow with 8 g N m^−2^ and 12 g N m^−2^ (Table 9). In rice after fallow rotation, rice yield showed a positive response to fertilizer rates even at 12 g N m^−2^, which suggested that higher yields could be produced with higher rates of N fertilizer application. In both years, legume crop residues incorporation in rice after winged bean and rice after long bean crop rotation systems was effective in producing a satisfactory yield even in 2011 when rice after winged bean was grown without N fertilizer. It was observed that rice grain yield decreased about 5–33% in the zero-N control among legume crop rotation in the second year of experiment. The rice grain yield proved that winged bean was more effective than N fertilizer for both years. Similar results were obtained in rice after hairy vetch with 4 or 8 g N m^−2^ [44]. In 2011, rice grain yield was slightly lower when grown without N fertilizer in the case of rice after winged bean rotation, but the addition of winged bean residues contributed noticeably to higher yield levels when compared with rice after fallow with 8 or 12 g N m^−2^. The present findings differed with observations from other studies [18], which have reported that rice yield did not increase or even decrease when rice straw residue was added without fertilizer N. The possible reason for this could be due to the use of rice straw residues under upland conditions which were not fully decomposed during the rice growing season. Additionally, grain yield of rice significantly increased when residues were incorporated into flooded soil [47]. Our results suggest that legume residues had a positive influence on both rice yield and N uptake when fertilizer was not used. Certainly, the influence of legume residues on rice yield depends on the soil nutrient status, texture, addition of organic matter, amount of residue returned to soil [33], and timing and levels of fertilizer N used.

Addition of legume residues and N fertilizer application had a significant effect on harvest indices (HI) for both years (Table 10). In both 2010 and 2011, rice crop rotation with winged bean grown with 4, 8, and 12 g N m^−2^, achieved higher HI compared to the other crop rotation systems. Rice after fallow or corn gown without fertilizer N recorded the lowest HI for both years. Rice after bush bean with 8 or 12 g N m^−2^ gave superior HI in 2010 and similarly rice after long bean or mung bean with 12 g N m^−2^ gave better HI in 2010. Rice after fallow or corn with 12 g N m^−2^ showed identical HI for both years (Table 10). The harvest indices were lower in all systems in 2011 except for rice after winged bean. It has been documented that harvest indices increases significantly with higher rates of N fertilizer application [55]. However, on the contrary, other studies have reported that with increasing N fertilizer, the harvest index decreased [56]. The lower HI with higher N fertilizer application suggested that N fertilizer influenced more biological yield than economic yield [57]. The grain harvest index values were 43% at zero N level and 50% at 400 mg N kg^−1^ across 19 upland rice genotypes [16]. Nitrogen significantly improved grain harvest index, nitrogen harvest index and plant height which are positively associated with grain yield [58, 59]. The higher levels of phosphorus (72 kg ha^−1^) application in rice also recorded higher harvest index [60]. Harvest index is a measure of success in partitioning assimilated photosynthate. An improvement of harvest index means an increase in the economic portion of the plant [61]. Our observations suggest that higher fertilizer N application and their residual effect along with N_2_ fixation from legume crops influence HI in legume crop rotation systems.

## 4. Conclusions

The present study has shown that both legume residues and N fertilizer affected grain yield, N uptake, NAE, and NRE, which were greater in 2010 than in 2011. Rice rotation by all legumes produced consistently higher grain yield and NUE than rice after fallow. However, the NUE declined with higher levels of fertilizer N used, reflecting poor N utilization by the rice crop. This indicated that though rice rotation with legume crops plays a significant role in the improvement of grain yield, higher levels can be sustained by compatible and proper management of residues and N fertilizer. The N difference method applied in this study exhibited that N produced by all the legumes was readily available and can be used efficiently by the rice crop. Winged bean was capable of producing greater amount of biomass and providing high quantities of total N, in addition to fixing substantial quantities of N. Without significant loss of yield level, winged bean plant residue incorporation can be an alternative source to N fertilizer for sustainable rice yield. However, the incorporation of long bean plant residues requires minimum N fertilizer (4 g N m^−2^) and can be an alternative to the sole use of N fertilizer while bush bean and mung bean plant residues along with N fertilizer (8 g N m^−2^) can also be an alternative to N management method to reduce N losses from N fertilizer applied to rice crop. Winged bean plant residues are able to provide sufficient N to the soil for the rice crop and afford an advantage equivalent to that of 4 to 8 g fertilizer N m^−2^, respectively. In conclusion, amongst the tested legumes, winged bean showed the greatest potential while the other legumes can also be used as a substitute or supplement in place of chemical/inorganic N fertilizers.

## Figures and Tables

**Table 1:** Biomass yield of bush bean, long bean, mung bean, winged bean, and corn as affected by nitrogen fertilizer.

Nitrogen		Biomass yield (g m^−2^)									
(g/m^2^)		Bush bean		Long bean		Mung bean		Winged bean		Corn*	
2010	2011	2010	2011	2010	2011	2010	2011	2010	2011	2010	2011
0 0*	0	148.2^c^	140.1^c^	138.4^c^	136.8^b^	134.9^c^	131.6^c^	179.8^b^	169.4^b^	462.5^d^	449.5^d^
2 4*	0	155.7^b^	149.8^b^	145.9^b^	140.7^b^	145.0^b^	141.7^b^	187.3^ab^	184.7^a^	537.5^c^	514.7^c^
4 8*	0	165.5^a^	156.4^a^	155.7^a^	149.2^a^	151.5^b^	148.2^b^	190.6^a^	187.6^a^	579.8^b^	560.3^b^
6 12*	0	170.0^a^	162.9^a^	161.9^a^	155.7^a^	158.3^a^	157.7^a^	197.2^a^	193.2^a^	635.2^a^	602.6^a^

Means followed by the same letters are not significantly different for each treatment mean (*P* < 0.05).

*Denotes N fertilizer rate for corn in 2010.

**Table 2:** Nitrogen uptake of bush bean, long bean, mung bean, winged bean, and corn as affected by nitrogen fertilizer.

Nitrogen		Nitrogen uptake (g m^−2^)									
(g/m^2^)		Bush bean		Long bean		Mung bean		Winged bean		Corn*	
2010	2011	2010	2011	2010	2011	2010	2011	2010	2011	2010	2011
0 0*	0	5.0^c^	4.7^c^	4.7^d^	4.6^c^	4.6^c^	4.5^d^	6.2^b^	5.6^b^	3.6^d^	3.4^d^
2 4*	0	5.3^b^	5.1^b^	5.0^c^	4.8^c^	5.0^b^	4.8^c^	6.5^ab^	6.3^a^	4.3^c^	4.0^c^
4 8*	0	5.7^a^	5.3^b^	5.4^b^	5.1^b^	5.2^b^	5.1^b^	6.7^a^	6.6^a^	4.8^b^	4.5^b^
6 12*	0	5.9^a^	5.6^a^	5.7^a^	5.4^a^	5.5^a^	5.3^a^	6.8^a^	6.9^a^	5.3^a^	4.9^a^

Means followed by the same letters are not significantly different for each treatment mean (*P* < 0.05).

*Denotes N fertilizer rate for corn in 2010.

**Table 3:** Nitrogen fixation of bush bean, long bean, mung bean, and winged bean as affected by nitrogen fertilizer.

Nitrogen		Nitrogen fixation (%)							
(g/m^2^)		Bush bean		Long bean		Mung bean		Winged bean	
2010	2011	2010	2011	2010	2011	2010	2011	2010	2011
0	0	29.4^a^	28.8^a^	24.4^a^	25.1^a^	22.1^a^	24.1^a^	42.1^a^	41.4^a^
2	0	19.2^b^	22.2^b^	14.2^b^	16.8^b^	13.3^b^	18.0^b^	33.4^b^	37.4^a^
4	0	16.5^c^	16.1^c^	11.9^c^	12.5^c^	9.2^c^	11.8^c^	29.4^b^	31.8^b^
6	0	9.9^d^	13.2^d^	5.8^d^	9.5^d^	3.1^d^	8.6^d^	21.7^c^	29.5^b^

Means followed by the same letters are not significantly different for each treatment mean (*P* < 0.05).

**Table 4:** Nitrogen recovery efficiency of bush bean, long bean, mung bean, and winged bean as affected by nitrogen fertilizer.

Nitrogen	Nitrogen recovery efficiency (%)							
(g/m^2^)	Bush bean		Long bean		Mung bean		Winged bean	
2010	2010	2011	2010	2011	2010	2011	2010	2011
0	0.0^c^	0.0^c^	0.0^c^	0.0^c^	0.0^c^	0.0^d^	0.0^d^	0.0^d^
2	14.0^b^	18.0^a^	15.0^b^	13.0^b^	19.5^a^	19.5^a^	15.5^a^	29.0^a^
4	16.3^a^	15.3^b^	17.0^a^	15.5^a^	16.5^b^	16.0^b^	14.5^b^	20.5^b^
6	14.8^b^	14.8^b^	15.7^b^	14.8^a^	15.7^b^	15.0^c^	11.2^c^	19.5^c^

Means followed by the same letters are not significantly different for each treatment mean (*P* < 0.05).

**Table 5:** Biomass yield of rice as affected by N fertilizer and legume residue.

N (g m^−2^)		Biomass yield (g m^−2^)					
		Rice after bush bean		Rice after long bean		Rice after mung bean	
2010	2011	2010	2011	2010	2011	2010	2011
0	0	986.0^c^	921.8^c^	948.5^c^	928.7^c^	952.8^c^	912.1^c^
4	0	1058.6^b^	993.5^b^	1069.4^b^	1037.5^b^	1066.1^b^	986.9^b^
8	0	1074.9^ab^	1074.9^a^	1135.5^ab^	1083.4^a^	1128.7^a^	1071.7^a^
12	0	1123.8^a^	1084.0^a^	1138.4^a^	1102.6^a^	1131.9^a^	1078.2^a^

N (g m^−2^)		Biomass yield (g m^−2^)					
		Rice after winged bean		Rice after corn		Rice after fallow	
2010	2011	2010	2011	2010	2011	2010	2011

0	0	1154.7^b^	1107.5^b^	905.5^d^	873.0^d^	941.4^c^	905.5^c^
4	0	1262.2^a^	1211.7^a^	931.6^c^	905.5^c^	1065.1^b^	993.5^b^
8	0	1283.4^a^	1244.3^a^	1026.1^b^	960.9^b^	1136.8^a^	1042.3^a^
12	0	1289.9^a^	1254.1^a^	1058.6^a^	1006.5^a^	1127.0^a^	1058.6^a^

Means followed by the same letters are not significantly different for each treatment mean (*P* < 0.05).

**Table 6:** Nitrogen uptake of rice as affected by N fertilizer and legume residue.

N (g m^−2^)		Nitrogen uptake (g m^−2^)					
		Rice after bush bean		Rice after long bean		Rice after mung bean	
2010	2011	2010	2011	2010	2011	2010	2011
0	0	9.4^d^	8.5^c^	9.3^c^	8.5^c^	9.1^c^	8.4^c^
4	0	10.4^c^	9.5^b^	10.4^b^	9.5^b^	10.2^b^	9.4^b^
8	0	11.0^b^	10.7^a^	11.5^a^	10.3^a^	11.4^a^	10.7^a^
12	0	11.6^a^	10.9^a^	11.6^a^	10.8^a^	11.5^a^	10.8^a^

N (g m^−2^)		Nitrogen uptake (g m^−2^)					
		Rice after winged bean		Rice after corn		Rice after fallow	
2010	2011	2010	2011	2010	2011	2010	2011

0	0	12.1^b^	11.3^b^	7.1^d^	6.8^d^	7.5^c^	7.1^d^
4	0	13.4^a^	12.5^a^	7.6^c^	7.2^c^	8.5^b^	7.9^c^
8	0	13.7^a^	12.8^a^	8.4^b^	7.7^b^	9.5^a^	8.4^b^
12	0	13.9^a^	12.9^a^	8.9^a^	8.3^a^	9.7^a^	8.7^a^

Means followed by the same letters are not significantly different for each treatment mean (*P* < 0.05).

**Table 7:** Nitrogen recovery efficiency of rice as affected by N fertilizer and legume residue.

N (g m^−2^)		Nitrogen recovery efficiency (%)					
		Rice after bush bean		Rice after long bean		Rice after mung bean	
2010	2011	2010	2011	2010	2011	2010	2011
0	0	0.0^c^	0.0^d^	0.0^c^	0.0^d^	0.0^c^	0.0^d^
4	0	28.8^a^	27.5^a^	28.8^a^	27.5^a^	27.5^a^	25.0^b^
8	0	29.5^a^	23.8^b^	29.4^a^	23.8^b^	28.8^a^	28.8^a^
12	0	19.6^b^	20.0^c^	19.6^b^	20.0^c^	20.0^b^	20.0^c^

N (g m^−2^)		Nitrogen recovery efficiency (%)					
		Rice after winged bean		Rice after corn		Rice after fallow	
2010	2011	2010	2011	2010	2011	2010	2011

0	0	0.0^d^	0.0^d^	0.0^c^	0.0^c^	0.0^c^	0.0^d^
4	0	32.5^a^	30.0^a^	10.0^b^	9.8^b^	25.5^a^	20.0^a^
8	0	20.0^b^	18.8^b^	16.3^a^	11.1^a^	25.0^a^	16.3^b^
12	0	15.0^c^	13.3^c^	15.0^a^	12.4^a^	18.3^b^	13.3^c^

Means followed by the same letters are not significantly different for each treatment mean (*P* < 0.05).

**Table 8:** Nitrogen agronomic efficiency (g g^−1^) of rice as affected by N fertilizer and legume residues.

N (g m^−2^)		Nitrogen agronomic efficiency (g g^−1^)					
		Rice after bush bean		Rice after long bean		Rice after mung bean	
2010	2011	2010	2011	2010	2011	2010	2011
0	0	0.0^d^	0.0^d^	0.0^d^	0.0^c^	0.0^d^	0.0^d^
4	0	13.0^a^	12.5^a^	21.2^a^	17.1^a^	14.8^a^	12.9^a^
8	0	10.7^b^	9.7^b^	15.1^b^	11.4^b^	12.6^b^	11.4^b^
12	0	7.4^c^	6.5^c^	12.8^c^	11.1^b^	8.3^c^	7.8^c^

N (g m^−2^)		Nitrogen agronomic efficiency (g g^−1^)					
		Rice after winged bean		Rice after corn		Rice after fallow	
2010	2011	2010	2011	2010	2011	2010	2011

0	0	0.0^d^	0.0^d^	0.0^c^	0.0^c^	0.0	0.0^d^
4	0	26.9^a^	23.6^a^	14.7^a^	16.3^a^	21.2^a^	13.8^b^
8	0	16.3^b^	14.7^b^	15.5^a^	14.3^b^	18.7^b^	15.1^a^
12	0	12.2^c^	12.5^c^	13.3^b^	12.2^b^	15.2^c^	11.4^c^

Means followed by the same letters are not significantly different for each treatment mean (*P* < 0.05).

**Table 9:** Grain yield of rice as affected by N fertilizer and legume residues.

N (g m^−2^)		Yield (g m^−2^)					
		Rice after bush bean		Rice after long bean		Rice after mung bean	
2010	2011	2010	2011	2010	2011	2010	2011
0	0	424.5^c^	358.3^c^	416.9^c^	403.9^c^	407.2^c^	395.7^c^
		(−21)	(−33)	(−22)	(−25)	(−24)	(−26)
4	0	495.1^b^	439.7^b^	501.6^b^	472.3^b^	495.1^b^	449.2^b^
		(−7)	(−18)	(−6)	(−12)	(−7)	(−16)
8	0	547.2^a^	472.3^ab^	537.5^a^	495.1^ab^	540.0^a^	488.7^a^
		(+2)	(−12)	(+1)	(−7)	(+1)	(−8)
12	0	553.7^a^	521.2^a^	570.0^a^	537.5^a^	565.3^a^	521.2^a^
		(+4)	(−2)	(+7)	(+1)	(+6)	(−2)

N (g m^−2^)		Yield (g m^−2^)					
		Rice after winged bean		Rice after corn		Rice after fallow	
2010	2011	2010	2011	2010	2011	2010	2011

0	0	537.5^b^	508.1^b^	348.5^d^	293.2^d^	371.3^c^	342.0^c^
		(+1)	(−5)	(−35)	(−45)	(−31)	(−36)
4	0	645.0^a^	602.6^a^	407.2^c^	358.3^c^	488.6^b^	423.5^b^
		(+21)	(+13)	(−24)	(−33)	(−8)	(−21)
8	0	667.8^a^	625.4^a^	472.3^b^	407.2^b^	534.2^a∗^	472.3^a^
		(+25)	(+17)	(−12)	(−24)	(100)	(−12)
12	0	684.0^a^	658.0^a^	508.1^a^	439.7^a^	553.7^a^	504.9^a^
		(+28)	(+23)	(−5)	(−18)	(+4)	(−5)

Means followed by the same letters are not significantly different for each treatment means (*P* < 0.05).

*Parenthesis values denote yield increase (+) or decrease (−) in percent; values are calculated based on rice after fallow with 8 g N m^−2^ (100%).

**Table 10:** Harvest indices of rice as affected by N fertilizer and legume residues.

N (g m^−2^)		Harvest indices					
		Rice after bush bean		Rice after long bean		Rice after mung bean	
2010	2011	2010	2011	2010	2011	2010	2011
0	0	42.9^c^	38.9^c^	43.5^c^	43.5^c^	42.7^c^	43.2^b^
4	0	46.8^b^	44.3^b^	45.6^b^	45.5^b^	46.4^b^	45.5^b^
8	0	50.9^a^	43.9^b^	45.7^b^	45.7^b^	47.6^b^	45.6^b^
12	0	49.3^a^	48.1^a^	48.7^a^	48.7^a^	49.5^a^	48.3^a^

N (g m^−2^)		Harvest indices (%)					
		Rice after winged bean		Rice after corn		Rice after fallow	
2010	2011	2010	2011	2010	2011	2010	2011

0	0	46.6^b^	45.9^b^	38.5^c^	33.2^c^	39.4^d^	35.3^c^
4	0	51.1^a^	49.7^a^	43.7^b^	39.6^b^	41.4^c^	37.7^b^
8	0	52.0^a^	50.3^a^	46.0^a^	42.4^a^	45.8^b^	42.2^a^
12	0	53.0^a^	52.5^a^	48.0^a^	43.7^a^	49.1^a^	43.1^a^

Means followed by the same letters are not significantly different for each treatment mean (*P* < 0.05).
